# Robust Design of an Optical Micromachine for an Ophthalmic Application †

**DOI:** 10.3390/mi7050085

**Published:** 2016-05-06

**Authors:** Ingo Sieber, Thomas Martin, Ulrich Gengenbach

**Affiliations:** Karlsruhe Institute of Technology, Institute for Applied Computer Science, Hermann-von-Helmholtz-Platz 1, 76344 Eggenstein-Leopoldshafen, Germany; thomas.martin@kit.edu (T.M.); ulrich.gengenbach@kit.edu (U.G.)

**Keywords:** robust design, tolerance analysis, freeform optics, optical modeling and simulation, design optimization, ophthalmic implant

## Abstract

This article describes an approach to the robust design of an optical micromachine consisting of a freeform optics, an amplification linkage, and an actuator. The robust design approach consists of monolithic integration principles to minimize assembly efforts and of an optimization of the functional components with respect to robustness against remaining assembly and manufacturing tolerances. The design approach presented involves the determination of the relevant tolerances arising from the domains manufacturing, assembly, and operation of the micromachine followed by a sensitivity analysis with the objective of identifying the worst offender. Subsequent to the above-described steps, an optimization of the functional design of the freeform optics with respect to a compensation of the effects of the tolerances is performed. The result leads to a robust design of the freeform optics and hence ensures a defined and optimal minimum performance of the micromachine in the presence of tolerances caused by the manufacturing processes and the operation of the micromachine. The micromachine under discussion is the tunable optics of an ophthalmic implant, an artificial accommodation system recently realized as a demonstration model at a scale of 2:1. The artificial accommodation system will be developed to replace the human crystalline lens in the case of a cataract.

## 1. Introduction

The design and realization of complex microoptical devices are challenging tasks since both fabrication tolerances of the individual optical components and assembly tolerances may severely impact the systems’ optical performance. There are two approaches to alleviating this challenge: Active alignment of the optical components and robust design. Active alignment of the optical components, on the one hand, is an expensive process and, on the other hand, often is not possible in miniaturized systems. The robust design approach is two-pronged. The first step on system level is to eliminate assembly steps as far as possible by applying monolithic integration principles. This implies selection of technologies that allow realization of the monolithic design. The technology selection in turn may have repercussions on the design of the optical components. With this step alone, a few assembly tolerances have been avoided. The second step on component level is to design the critical components in such a way that their optical performance becomes robust *i.e.*, as invariant as possible against the remaining tolerances. This robust design approach shall be illustrated by the example of a demonstration model of an ophthalmic implant.

The Artificial Accommodation System (AAS) is a highly integrated system *i.e.*, an ophthalmic device designed for restoration of the accommodation ability of the human eye [1]. It is a mechatronic microsystem designed to be implanted into the capsular bag of the human eye [2] and consists of five functional units. The active optical unit *i.e.*, the optical micromachine, is composed of a freeform optics, an amplification linkage, and an actuator to form a tunable optical unit [3,4]. A further unit addresses the sensing and the control of the accommodation [5,6]. The internal and external communication of the implant is provided by an additional unit [7]. The units are integrated in a housing [8] and an energy supply [9] covers the energy demand of the implant.

**Figure 1:**
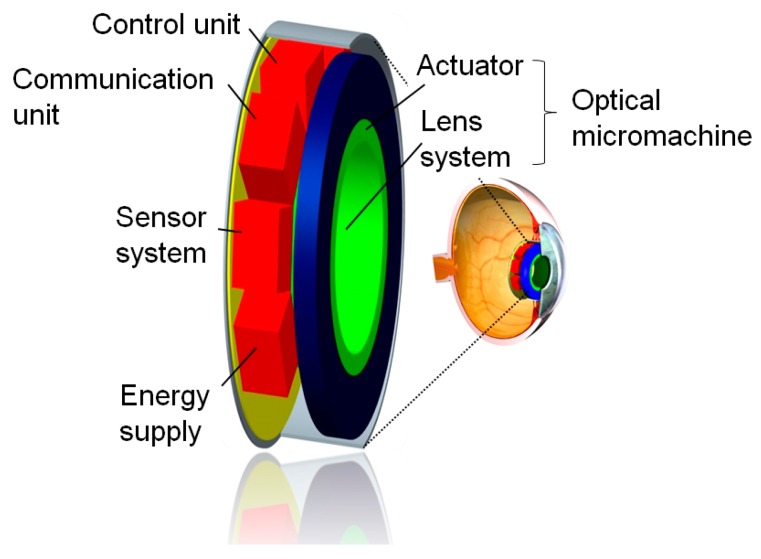
Schematic representation of the AAS and its position within the capsular bag of the human eye (inset on the right).

The components of the AAS are packaged in a glass housing with integrated optical surfaces. A demonstration model of this eye implant was designed and realized at a scale of 2:1 [10]. The optical subsystem of the demonstrator is composed of the glass housing with integrated optics and the optical micromachine [11]. Figure 1 shows a schematic representation of the AAS with its main components. The position of the implanted system within the capsular bag of the human eye is depicted also.

The set-up of the AAS is realized in a modular way so that different types of active optical subsystem can be integrated [12]. For analysis and evaluation of different optical principles for the adjustment of refraction power, an axially moveable optics as well as a laterally moveable optics can be mounted in the housing. The optics integrated in the housing are designed for use of an axially moveable lens. To be able to analyze and evaluate laterally moveable optics with only one housing design, an inset lens is used to provide the necessary basic refraction power. The inset lens is fixed by an index-matched adhesive to the optics integrated in the rear window of the housing. The rear surface curvature matches the curvature of the optics integrated. The mechanical interface for mounting the different types of optics into the housing has been designed to allow for that modularity. The reference for positioning of the optical subsystem is the rear wall of the glass housing. Both types of optical subsystems are mounted to this surface by means of an ultraprecision milled mount.

The optical micromachine is of vital importance to the total system since the optical performance of the AAS depends on its reliable function. For this reason the above-outlined two-pronged robust design approach was applied.

The article is organized as follows: Section 2 presents the optical subsystem of the AAS. Section 3 covers the determination of the relevant tolerances of the optical subsystem and the tolerance analysis of the optical micromachine based on a sensitivity approach. The optimization of the freeform optics with the objective of minimizing the impact of the tolerances on the systems’ performance as well as a discussion of the results are presented in Section 4. The paper is completed by a discussion of the presented robust design approach.

## 2. Optical Subsystem of the Demonstration Model

The components of the optical subsystem of the demonstration model are comprised of the optical micromachine, a glass housing with optics integrated, and an additional inset lens fixed by an index-matched adhesive to the optics integrated in the rear window of the housing.

### 2.1. Optical Micromachine

The optical micromachine consists of three principal components freeform optics, actuator, and amplification linkage. The following subsections present and discuss the individual principal components.

#### 2.1.1. Optics

**Figure 2:**
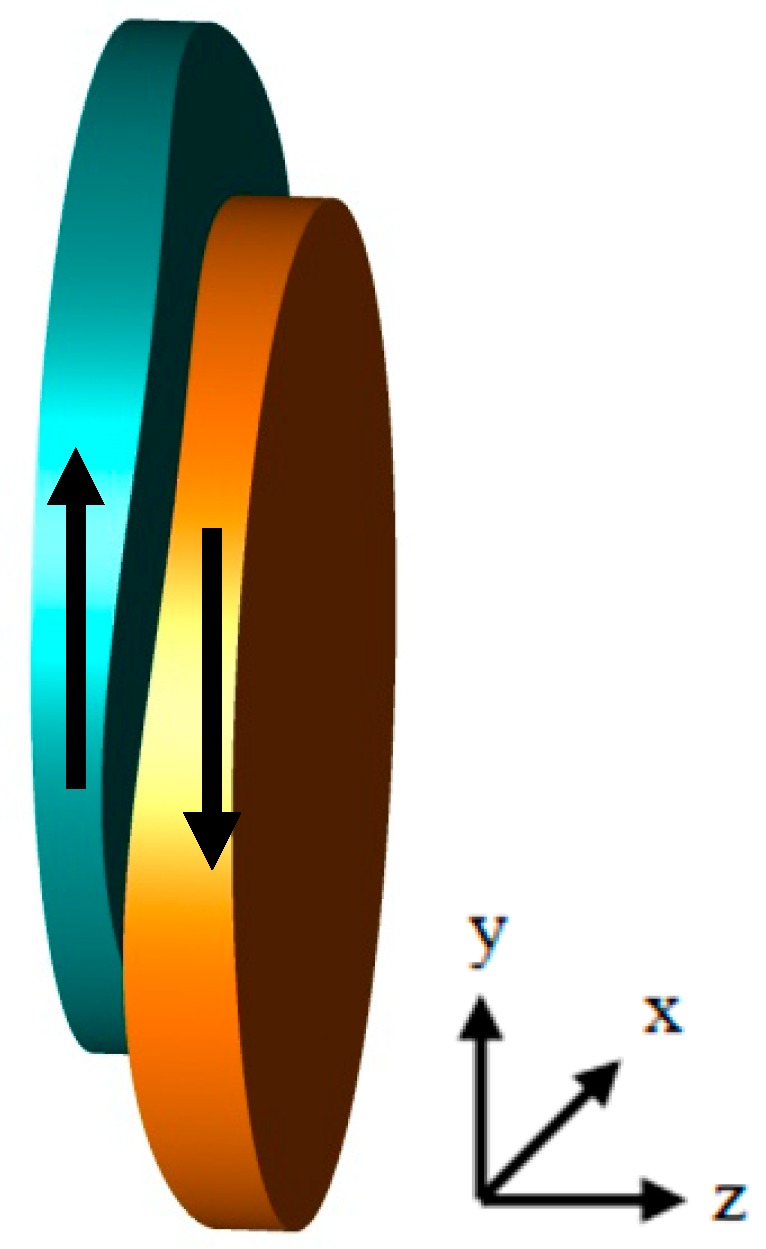
Alvarez-Humphrey optics. Variation of the refraction power by means of a shift of the Alvarez-Humphrey surfaces perpendicular to the optical axis (*z*-axis in the figure).

The optical concept discussed in this article works according to the principle invented in the 1960’s by Alvarez and Humphrey [13]. According to this principle, in an optical system comprising of two cubic-type lens parts the refraction power is varied by mutually shifting both lens parts laterally as shown in Figure 2. AH lenses were studied extensively [14,15,16] and proposed for different technical [17,18,19] as well as ophthalmological applications [20,21].

The opposing surfaces are conjugated and of a cubic shape. Parameters for designing Alvarez-Humphrey (AH) lenses are the lateral movement *v* of the lens parts and the “form” parameter *A* determining the shape of the lens surface. The polynomial description of the surface sag is given by Equation (1):

*z*(*x*,*y*) = *A*((*y − v*)*x*^2^ + 1/3(*y − v*)^3^) (1)

Lateral movement of lens parts is advantageous in all applications, where the space available along the optical axis is limited, as is true for the demonstration model at hand. Requirements of the optics and its design are presented in [22]. To compensate for aberration effects and fulfill the requirements of the demonstrator’s specifications, the description of the AH surfaces is expanded and is represented by a polynomial of fourth order (Equation (2)). The parameters used are depicted in Table 1. The center thicknesses of the individual lens parts are 500 µm, material used was polymethyl metacrylate (PMMA) with a refractive index of *n*_D_ = 1.4905.

*z*(*x,y*) *= a*_1_*x*^4^ + *a*_2_*x*^3^*y* + *a*_3_*x*^2^*y*^2^ + *a*_4_*xy*^3^ + *a*_5_*y*^4^ + *a*_6_*x*^3^ + *a*_7_*x*^2^*y* + *a*_8_*xy*^2^ + *a*_9_*y*^3^ + *a*_10_*x*^2^ + *a*_11_*xy* + *a*_12_*y*^2^ + *a*_13_*x* + *a*_14_*y* (2)

**Figure 3:**
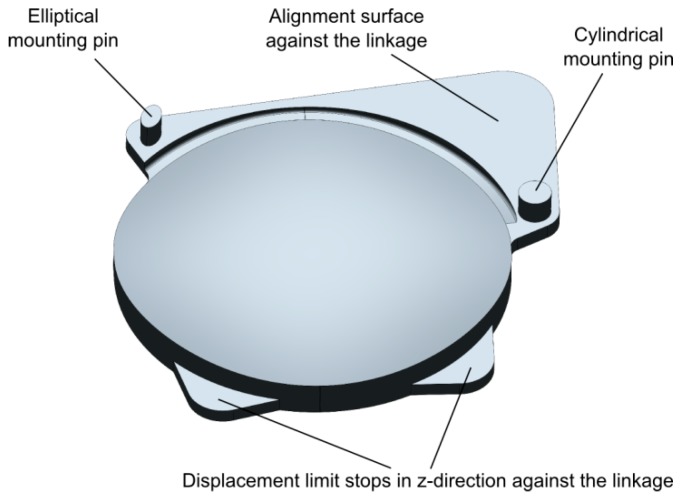
Computer Aided Design (CAD) model of one of both identical lens parts of the AH optics with alignment structures indicated [1].

The performance of AH optics depends crucially on a proper alignment of the two lens parts [23]. Alignment of both identical lens parts to the linkage in *x*- and *y*-direction is realized by two mounting pins and an alignment surface (see Figure 3). The mating features of the amplification linkage are two cylindrical holes and the respective planar alignment surface on the output stage. Alignment in *z*-direction is accomplished by the two planar contact surfaces. The alignment surfaces are enlarged to reduce rotation/tilt around an axis through both pins. The position of the mounting pins on the lens parts is subject to high tolerances due to shrinkage during the injection molding process. In order to avoid over-determining by mating two cylindrical pins with two cylindrical holes a statically determinate pin arrangement was selected. A cylindrical mounting pin defines the *x*,*y* position of the lens part in the plane, while the second pin with an elliptical shape defines the remaining rotational degree of freedom. This way positional tolerances between the cylindrical holes in the silicon amplification linkage and the injection molded pins of the lens parts can be compensated.

The freeform optics was manufactured using microinjection molding, which is a replication process with the potential for mass fabrication. This process also allows for monolithic integration of adjustment and alignment structures and optical surfaces to optimize positioning accuracy in assembly. To prepare manufacture, a set of simulation experiments as well as process optimization were conducted to demonstrate the feasibility of freeform surface fabrication by means of a microinjection molding process [24]. One result of these simulations was that all cavities, including the mounting pins of the integrated fixing structures, and the thinnest part of the lens with a thickness of 100 µm can be completely filled [25]. On the basis of this result, the molding tool was fabricated and the freeform optics was molded under consideration of the optimized parameter set.

Adaptation of the refraction power of the optics to the accommodation demand is done by a piezoelectric stack actuator and a silicon linkage for transmission and amplification of the actuation stroke [26].

#### 2.1.2. Actuator

The displacement-specific change of the refractive power of a lateral-shift optics can be designed in a wide range. However, for cost-effective fabrication, a trade-off between a small lens displacement to be provided by the actuator and high robustness of the optics against deviations of shape and position of the optical surfaces must be made. A relative displacement difference of both lens parts of 360 µm with the ideal actuator rest position being at 81 µm relative lens displacement allows for a change of 4.6 dpt in optical power and was found to be a reasonable compromise for the demonstration model [27]. The requirements of the actuator with respect to cycle lifetime, power consumption, fail-safe behavior, response time, and space can be found in [4]. The initial actuator geometry was optimized to fit into a micromachine with a cylindrical design space. As the availability of this actuator became an issue, an oversized commercial actuator had to be selected. Hence, the scaled cylindrical design space of the micromachine had to be violated locally and the design had to be adapted accordingly. The final actuator for the demonstration model was a piezoelectric stack actuator (Noliac A/S, NAC2001-H06, Noliac, Kvistgaard, Denmark) with a nominal stroke of 4.9 µm of which a maximum of 2.9 µm are used during operation [26].

#### 2.1.3. Amplification Linkage

**Figure 4:**
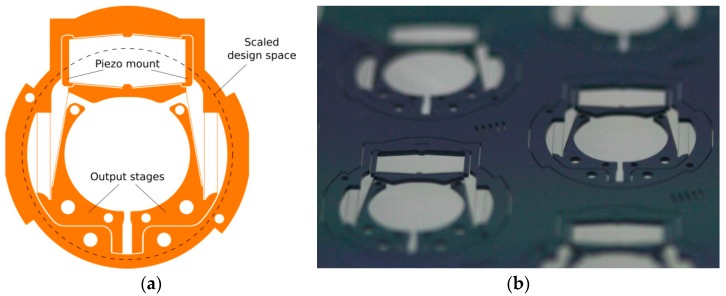
Design of the amplification linkage (**a**) and realized silicon linkages manufactured by DRIE (**b**).

To provide the maximum relative lens part displacement difference of 279 µm calculated from the actuator rest position (*i.e.*, a stroke of about 140 µm per lens part), a displacement amplification ratio of at least 48.1 is required. For reasons of miniaturization and reduction of assembly processes, a monolithic design with flexure hinges was selected. The planarity of the linkage enables a Deep Reactive Ion Etching (DRIE) process for fabrication of the linkage in single-crystal silicon [4]. Figure 4 depicts the linkage as designed (left) and the linkage manufactured by DRIE (right) fulfilling the transmission requirements [26]. The trapezoid-shaped structure in the upper part of the linkage serves as a mount for the stack actuator. Self-aligning structures to mount the lens parts as well as limit stop structures for overload protection were integrated in the output stages of the linkage. The minimum structure width in the silicon linkages is 24 μm. The linkage is enclosed in a solid frame that serves both as interface to the subsystem mount and as handling frame during subsystem assembly.

#### 2.1.4. Set-up of the Optical Micromachine

**Figure 5:**
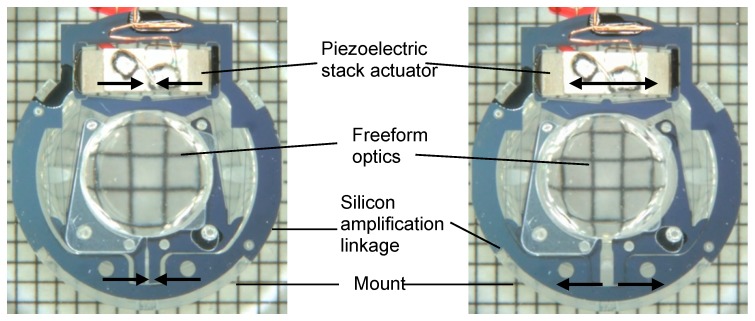
Optical micromachine and its components: Tunable freeform optics, silicon linkage, and piezoelectric stack actuator [1]. **Left**: contracted actuator and accommodated state, **right**: expanded actuator and disaccommodated state of the optics.

Assembly of the freeform optics, the piezo stack actuator, and the silicon linkage yields the optical micromachine. Figure 5 shows two photographs of the optical micromachine in two different actuation states. On the left hand side, the piezo stack actuator is contracted leading to an inward movement of the lens parts and the accommodated state, while on the right the actuator is in an expanded state leading to an outward movement of the two lens parts and therefore to the disaccommodated state. Also shown in Figure 5 is the mount, which is the mechanical interface to the glass housing.

### 2.2. Additional Optical Components

As mentioned above, the AAS features a modular concept, where housing as well as optical surfaces integrated in the housing will be used for different optical principles. Originally, the housing was designed for an optical micromachine using an axially moveable lens. Hence, the rear-side optics is a negative aspheric lens. Combining this rear-side optics with an AH optics, an aspherical inset lens has to be designed to add a positive refraction power to the negative lens of the rear side. The inset lens is monolithically integrated with the diamond-lathed mount that forms the interface between active optics subsystem and glass housing. Since the rear surface of the asphere has to match the curvature of the optics integrated in the housing, only the aspheric front surface remains as free design parameter for beam modification. The inset lens was manufactured from PMMA with a refractive index of *n*_D_ = 1.4905.

The sag of the aspheric front surface of the inset lens is given by Equation (3), with *r*^2^ *= x*^2^ *+ y*^2^, the conic constant *k*, and the curvature *c* (reciprocal value of radius). The center thickness of the inset lens is 1.6 mm.

(3) $$z=\frac{c\;r^{2}}{1+\sqrt{1-\left(1+k\right)c^{2}\;r^{2}}}+\beta_{1}\;r+\beta_{2}\;r^{2}+\beta_{3}\;r^{3}+\beta_{4}\;r^{4}+\beta_{5}\;r^{5}+\beta_{6}\;r^{6}+\beta_{7}\;r^{7}+\beta_{8}\;r^{8}$$

The parameters used are depicted in Table 2.

The optical surface integrated in the front half shell of the housing is also an asphere with a center thickness of 250 µm, whose surface sag is described by Equation (4). The material used for precision molding of the half shells was the Schott glass BF33 with a refraction index of *n*_D_ *=* 1.4714.

(4) $$z=\frac{c\;r^{2}}{1+\sqrt{1-\left(1+k\right)c^{2}\;r^{2}}}+\beta_{2}\;r^{2}+\beta_{4}\;r^{4}+\beta_{6}\;r^{6}$$

The parameters used are shown in Table 3.

As a substitute of the human cornea a commercially available converging lens was used *i.e.*, the NT 47-873 lens supplied by Edmund Optics (Barrington, NJ, USA) with an effective focal length of 22.5 mm [28].

**Figure 6:**
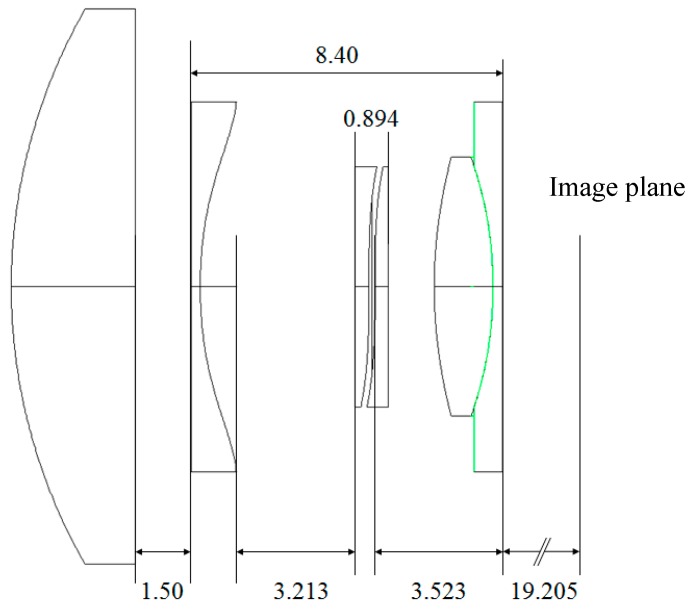
Optical model of the AAS.

Figure 6 shows a sectional view of the optical model of the AAS with the remaining geometrical parameters given. The boundary between the inset lens and the optics integrated in the housing is shown in green.

### 2.3. Set-up and Model of the Optical Subsystem

**Figure 7:**
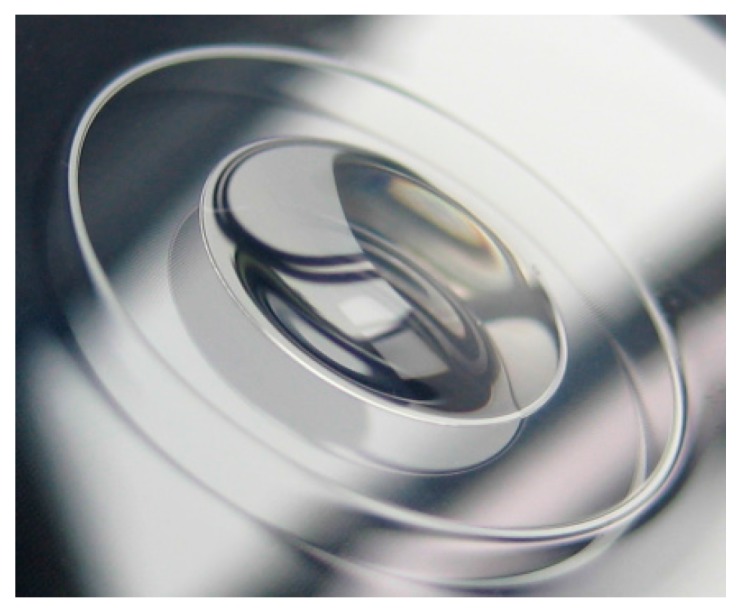
Precision-molded rear window of the demonstrator with its integrated optics (optical aperture of 6 mm) [1].

**Figure 8:**
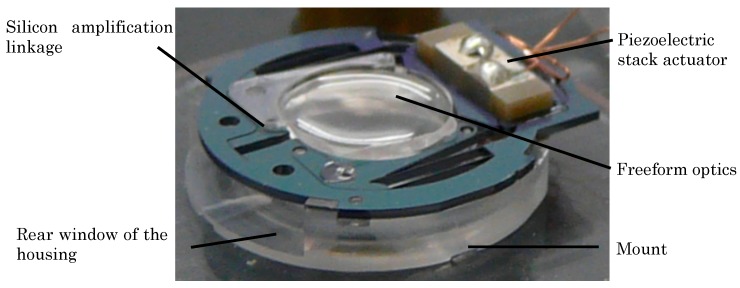
Optical micromachine assembled to the rear half shell of the glass housing.

The optical subsystem of the demonstrator is composed of a glass housing with integrated optics, the inset lens and the optical micromachine. The housing consists of two half shells containing a shaped entrance and exit window providing the basic refraction power of the optical system [11]. A precision molding process was chosen for fabrication of the entrance and exit window of the glass housing. Figure 7 shows a photograph of the precision-molded rear half shell and an integrated optics with an aperture of 6 mm. Figure 8 shows the optical micromachine assembled on its mount in the rear half shell of the glass housing.

**Figure 9:**
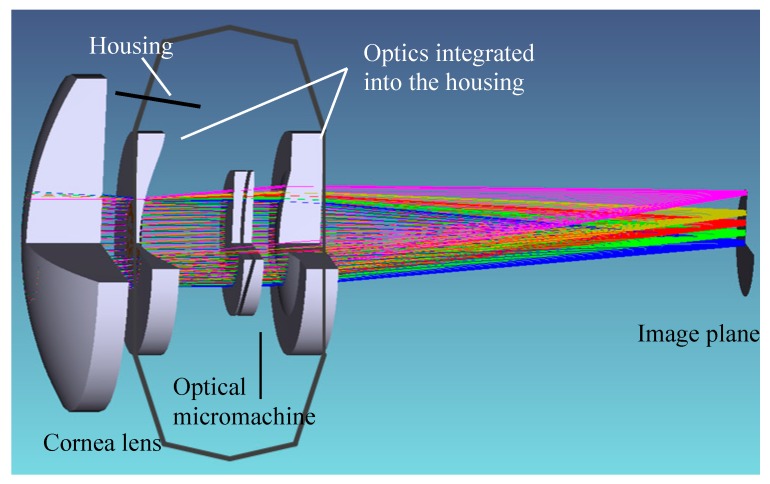
Model of the demonstrator optics of the AAS: Cornea lens, sketch of the housing with the integrated optics, the optical micromachine, and the image plane [29].

To be able to conduct a tolerance analysis simulation, a model of the entire optics has to be generated. Figure 9 shows the optical model of the subsystem with some calculated rays in the case of four fields spanning half of the field angle of the foveal view of the human eye and a sketch of the housing. The optical model consists of the optical subsystem as defined above and an additional lens representing the cornea of the human eye.

## 3. Tolerance Analysis

The above-defined model was used to conduct a sensitivity analysis with respect to the manufacturing and operational tolerances. The first step in this approach is to determine the relevant tolerances.

### 3.1. Determination of the Relevant Tolerances

Tuning of the refraction power of the demonstration model is achieved by a lateral shift of the two AH lens parts relative to each other in *x*-direction. Consequently, the focus in tolerance analysis of the optical subsystem lies on the AH optics. To obtain a good optical performance, two points are critical, namely manufacturing accuracy of the optical freeform surfaces and a proper alignment of the freeform surfaces in the static as well as in the dynamic state.

Effects of manufacturing tolerances of the freeform optics were investigated and are described in [22]. The results of this tolerance analysis show only negligible differences in the performance of the freeform surfaces manufactured by means of the microinjection molding process (represented by their metrology data) and the nominal surfaces.

Besides the manufacturing accuracy of the freeform surfaces, a proper alignment of the surfaces to each other is of fundamental importance. A positioning error of the two lens parts may have a severe impact on the performance of the total system [30]. The tunable optics is guided and shifted by a deformation of the monolithic silicon linkage. Hence, it is of great interest to determine the impact of mispositioning induced by assembling the lens parts and the silicon linkage. Mispositioning of the lens parts relative to the nominal positions can have three different origins. The first of which are the manufacturing tolerances. Fabrication of the silicon linkage as well as of the positioning structures and the optical surface integrated in the freeform optics is subject to tolerances. Then tolerances related to the assembly process have to be taken into account. Here position errors in joining the lens parts and the linkage and in joining the linkage with the mount and the housing play a crucial role and have to be considered. Last but not least operational influences can lead to tolerances. Operational influences are primarily caused by gravitational forces on the lens suspensions. Furthermore imperfections of the deformation kinematics of the linkage lead to a mispositioning of the lens parts relative to the nominal positions.

**Figure 10:**
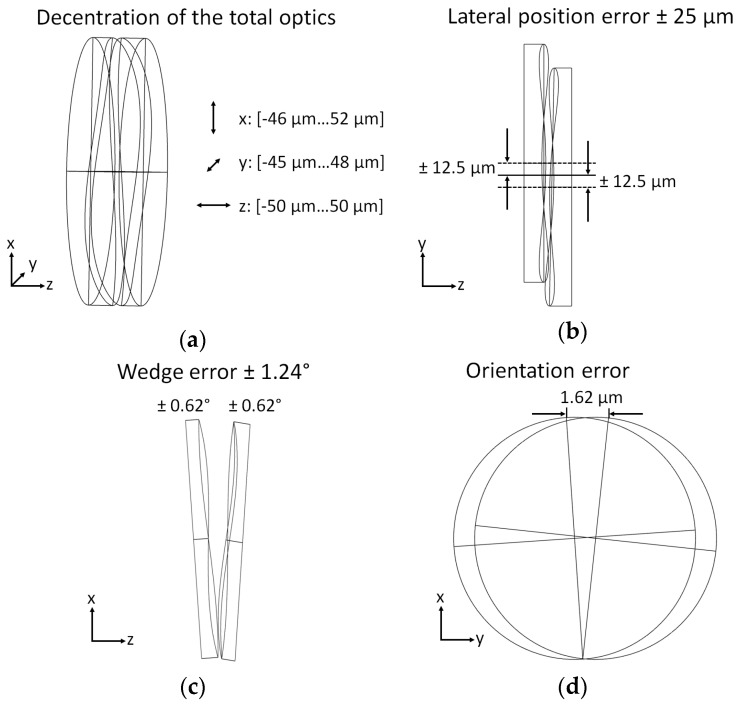
Different types of position errors identified for the AH optics.

These tolerances and influences cause a positioning error of the freeform surfaces. Four main types of positioning errors were identified (see Figure 10). A decentration of the total optics with respect to the glass housing has been significantly reduced due to monolithic integration of the inset lens with the mount for the micromachine. The remaining tolerances result from the assembly of the mount into the housing, which is facilitated by the self-alignment of the inset lens in the matching concave shape of the rear glass housing. In the next assembly step the optical micromachine in turn is aligned on the mount by specifically designed matching alignment structures on the upper rim of the mount and in the handling frame of the amplification linkage. The DRIE process for fabrication of the linkages does not yield perfectly vertical sidewalls. Thus the intended cylindrical holes turned out to have a slight conicity. The diameter tolerance of the cylindrical holes on the wafer front side was 800 ± 2 µm, while the backside tolerance of the hole was 800 + 40 µm. In order to allow insertion of the mounting pins of the lens into the silicon linkage during assembly a 15 µm clearance between nominal hole diameter and nominal pin diameter was selected yielding a nominal mounting pin diameter of 795 µm. Shrinkage of PMMA in injection molding is typically in the range of 0.1%–0.8% and within that range severely influenced by injection molding process parameters (holding, packing pressure, mold cooling). On the basis of these individual tolerances the remaining position errors have been estimated (see Table 4).

The lateral position error means a relative displacement of both lens parts perpendicular to the functional shifting direction. This is caused by assembly tolerances between lens parts and linkage as well as by manufacturing tolerances of the lens parts and is estimated to be in the range of −25 µm–+25 µm. The wedge error is caused by a relative rotation of both lens parts around an axis perpendicular to the optical axis and the shift axis, due to assembly tolerances between lens parts and linkage and is estimated to be in the range of −1.24°–+1.24°.

The cause of the orientation error is a relative rotation of both lens parts around an axis parallel to the optical axis. In [31], it was shown that the orientation error may have a severe impact on the system’s performance. The orientation error can be reduced drastically by a proper design of the alignment structures. In our case, it is considered to be negligible compared to manufacturing and assembly tolerances. Operational influences caused by gravitational forces and the imperfect deformational kinematics of the linkage remain as error sources. Mechanical simulations of the actuator system resulted in an orientation error range of −0.003°–+0.012°.

### 3.2. Sensitivity Analysis of the Optical Micromachine

**Figure 11:**
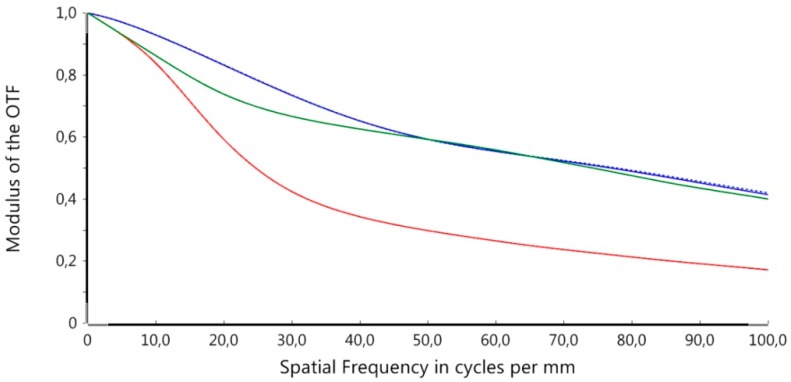
MTF of the nominal configuration and three different refraction power adjustments: −0.8 dpt (red), 1.5 dpt (blue), and 3.0 dpt (green).

For investigating the influence of the individual tolerances on an evaluation criterion and to find the worst offenders, a sensitivity analysis was conducted. The modulation transfer function (MTF), a widely accepted measure for optical imaging quality, was used as evaluation criterion [31]. Figure 11 shows the MTF of the nominal (error-free) configuration for three different adjustments of refraction power, in particular −0.8 dpt (far vision, shown in red), 1.5 dpt (intermediate vision, shown in blue), and 3.0 dpt (near vision, shown in green). The MTF was evaluated up to a limit of 100 cycl./mm. Noticeable is the decrease of the MTF in case of the far vision adjustment. This is due to the hyperopic design of the optics to be able to adapt the refraction power range of the optics to the patient’s demand after implantation.

**Figure 12:**
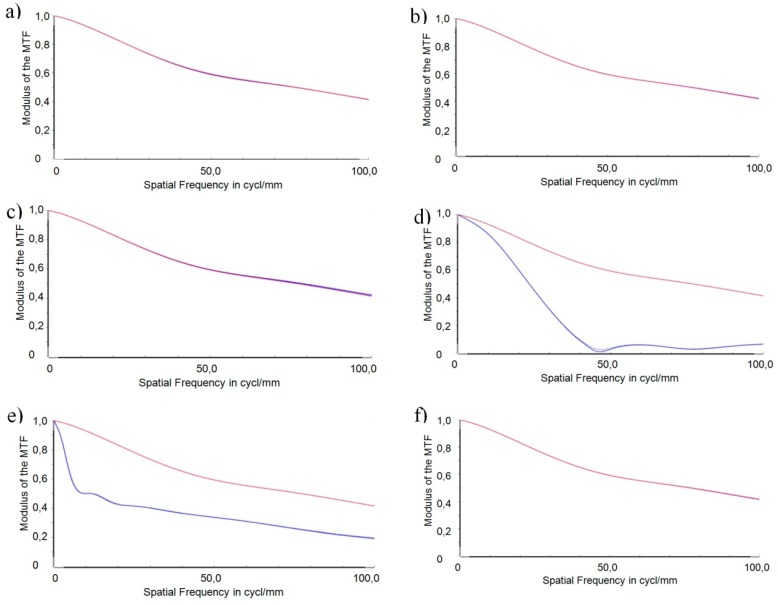
MTFs for the different types of position tolerances identified (blue) in comparison to the reference (red). (**a**) Decentration in *x*; (**b**) Decentration in *y*; (**c**) Decentration in *z*; (**d**) Lateral position error; (**e**) Wedge error; (**f**) Orientation error.

For better clarity, the following presentation is limited to the MTF of the central field and an adjustment of the accommodation state of 1.5 dpt. In this sensitivity analysis the worst case values of the individual tolerances were chosen to calculate the MTF. The simulations were done with the optical simulation tool OpticStudio 15.5 (ZEMAX LLC, Kirkland, WA, USA) [32]. Figure 12 depicts the simulation results of the MTF for the four main position errors identified in a worst-case configuration (drawn in blue). The error of decentration of the total optics was calculated for the three directions *x*, *y*, and *z*. The MTF of the nominal (error-free) configuration is the reference for the tolerance analysis and is depicted as a red curve in the following graphs.

Evaluating the effects of the different position errors on systems performance leads to the following results.

A comparison of the decentration of the total optics in *x*-, *y*-, and *z*-direction, respectively (Figure 12a–c) with the reference does not reveal any relevant impact of this position error on the imaging quality of the optical subsystem. Hence, it may be concluded that the optical subsystem is robust with respect to a decentration of the complete optics in the specified range.

Consideration of the lateral position error (Figure 12d) reveals that the displacement of the two lens parts against each other perpendicular to the functional shifting direction has a severe impact on the imaging quality of the optical subsystem. The MTF drops steeply and meets the *x*-axis at 45 cycles per mm. A spatial resolution above this value is not possible anymore *i.e.*, the lateral position error in its worst case configuration limits the spatial resolution drastically from 0.42 at 100 cycl./mm to 0.0 at 45 cycl./mm.

Tilting both surfaces perpendicular to the optical axes, as described by the wedge error (Figure 12e), leads to a steep decrease of the MTF curve in the lower frequency range below 10 cycl./mm. Above 10 cycl./mm, the slope of the decrease is only small and results in an MTF value of 0.2 at a spatial frequency of 100 cycl./mm.

In the worst case configuration of the orientation error, the axis of rotation lies parallel to the optical axis at the lower edge of the optics (as shown in Figure 10). The rotation leads to a lateral displacement of all points on the surface with a distance to the axis of rotation. At the upper edge of the optics, the lateral displacement amounts to 1.62 µm. No relevant impact of the orientation error on the imaging quality can be seen (Figure 12f).

One result of the tolerance analysis performed is that the set-up of the demonstrator of the AAS is robust with respect to a decentration of the total optics. The impact of the orientation error is negligible due to precautions taken in the design of the alignment structures. Comparison of the position errors of the individual freeform surfaces yields the following findings: Wedge error and lateral position error will have very severe impacts on the imaging quality of the demonstrator. In the case of the lateral position error the resolution of spatial frequencies is limited to 45 cycl./mm. Lateral displacement along an axis perpendicular to the shift axis of the AH freeform optics was identified to be the worst offender in this sensitivity analysis. This lateral displacement is the core of the lateral position error. An optimization of the design of the freeform optics in terms of robustness to reduce the impact of the lateral position error on the system’s performance was conducted and is presented in the following section.

## 4. Optimization and Results

The optimization with respect to a robust design was conducted with an actively damped least squares algorithm provided by the optical simulation software OpticStudio 15.5. The local search algorithm is capable of optimizing a merit function composed of weighted target values. Optimization parameters *i.e.*, parameters to be changed by the algorithm are the parameters describing the shapes of the individual freeform surfaces. Using this approach, the shapes of the freeform surfaces can be altered independently of each other, hence both surfaces are not mandatorily conjugated anymore. Only by this measure and an additional extension of the polynomial description relevant improvements could be reached. Additionally, the parameters of the aspheric front surface of the inset lens described by Equation 3 are used as parameters for optimization. The modular concept of the optical set-up is not afflicted by this. As a criterion to control the optimization the MTF was calculated at different sampling points with individual weighting factors. Geometrical and manufacturing restrictions were used to formulate the boundary conditions to ensure that the optimization results in a reasonable design.

**Figure 13:**
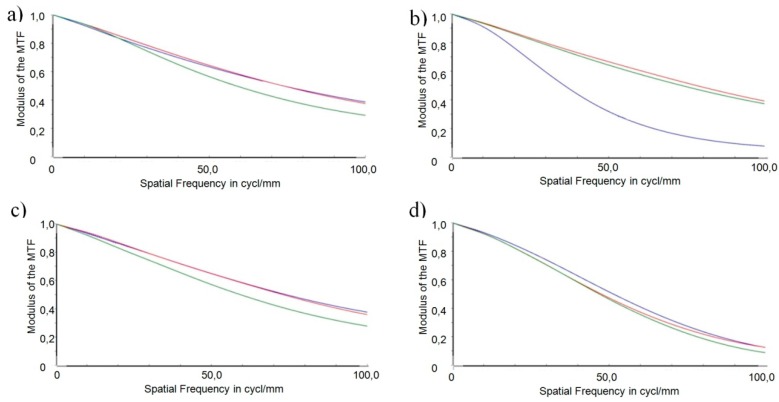

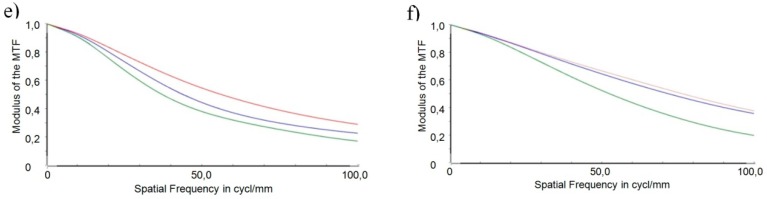
MTF for the different types of position tolerances identified and the robust design for three different adjustments of refraction power: −0.8 dpt (red), 1.5 dpt (blue), and 3.0 dpt (green). (**a**) Decentration in *x*; (**b**) Decentration in *y*; (**c**) Decentration in *z*; (**d**) Lateral position error; (**e**) Wedge error; (**f**) Orientation error.

Figure 13 shows the MTFs of the individual position tolerances for three different adjustments of refraction power: −0.8 dpt (red), 1.5 dpt (blue), and 3.0 dpt (green) using the optimized freeform surfaces.

Figure 13 illustrates that the optimized robust design performs relatively even between the distinct adjustments of refraction power. The only exception is the decentration in *y*, where the MTF for the intermediate vision experiences a strong degradation compared to the two other adjustments. The even performance is owed to the fact that the surfaces of both AH lens parts were optimized independently of each other.

**Figure 14:**
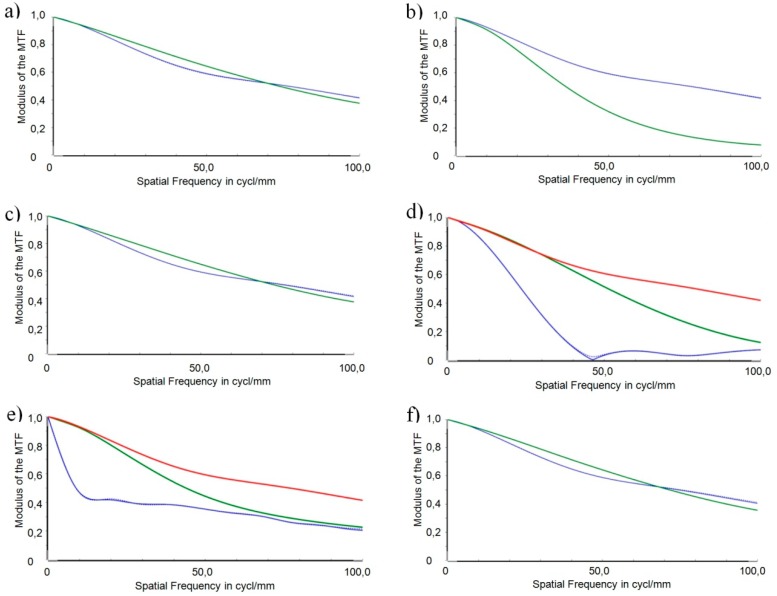
MTF for the different types of position tolerances identified and the robust design (green) in comparison with that of the original design as shown in Figure 12 (blue), and the MTF of the error-free configuration (red). (**a**) Decentration in *x*; (**b**) Decentration in *y*; (**c**) Decentration in *z*; (**d**) Lateral position error; (**e**) Wedge error; (**f**) Orientation error.

Again, for better clarity, Figure 14 only shows the MTFs in the case of the intermediate vision (1.5 dpt). Figure 14 depicts the individual position tolerances using the optimized surface geometries (green) in comparison to the original design as shown in Figure 12 (blue). The reference for evaluation of the robust design is the nominal (error-free) MTF, which is depicted in the graphs as a red curve.

It can be stated in general that the severe impacts of the position errors *lateral position error* (Figure 14d) and *wedge error* (Figure 14e) are fixed by the robust design. This improvement is at the expense of a performance degradation in the case of the decentration error in *y* (Figure 14b). In the following, a comparison of the robust design (green) with the original design (blue) is conducted for the individual position errors.

Concerning the decentration of the total optics in *x*-, *y*-, and *z*-direction, respectively (Figure 14a–c), the reference MTF coincides with the MTF resulting from the original design (see also Figure 12 a–c). A slight improvement of the MTF resulting from the robust design can be stated in the frequency range 10 cycl./mm to up to 70 cycl./mm for the decentration errors in *x* and *z* in comparison with the reference. The MTF curves intersect at 70 cycl./mm. For frequencies above 70 cycl./mm, the robust design achieves marginally lower values compared with the original design (and the reference). In the case of a decentration in *y*, the robust design is more sensitive, the course of its MTF curve is smooth and without steep drops but runs below that of the original design over the complete frequency range shown.

The lateral position error (Figure 14d) had the most severe impact on the optical performance for the original design. The improvement of the robust design approach is most obvious in this case. The collapse of the MTF at 45 cycl./mm is fixed and the resulting MTF curve is smooth in the higher frequencies. Nevertheless, the MTF curve resulting from the robust design runs below the reference curve beyond 40 cycl./mm.

Concerning the wedge error (Figure 14e), the steep decrease in the low-frequency range is obliterated by the robust design. In the higher frequency range, both curves approach each other. Again, the MTF resulting from the robust design runs below the reference curve for frequency values beyond 10 cycl./mm.

In the case of the orientation error (Figure 14f), the reference MTF coincides with the MTF resulting from the original design (see also Figure 12f). The course of the curves resembles that of the decentration errors in *x* and *z*: The performance of the robustly designed optics is comparable to that of the original design (and the error-free design), with slightly higher values below and marginally lower values above 70 cycl./mm.

**Figure 15:**
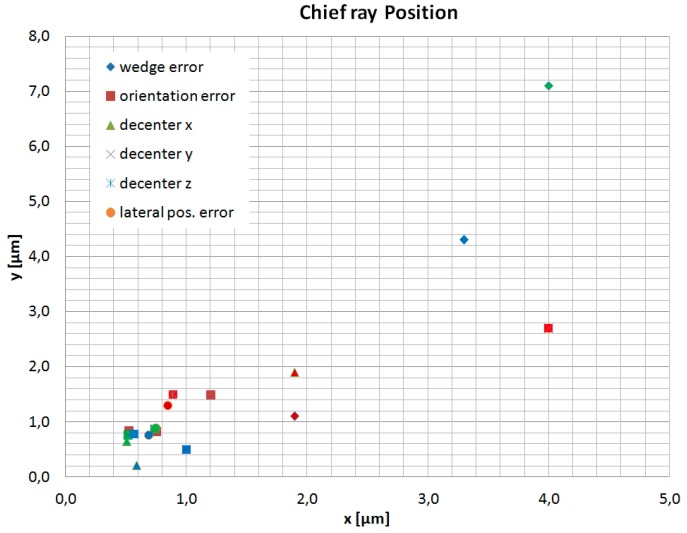
Chief ray positions for the individual position errors of the freeform optics for three different adjustments of refraction power: −0.8 dpt (red), 1.5 dpt (blue), and 3.0 dpt (green).

As expected, the optimization with respect to a robust design results in two different descriptions of the freeform surfaces violating the conjugation of the originally designed AH optics. Furthermore, parameters of higher order of the polynomial equation describing the freeform surfaces received significant values. The mathematical description of the sag of the optimized freeform surfaces as well as the parameters of the individual surfaces are given in Appendix A. The robust freeform surfaces are described by a seventh order polynomial equation using 35 parameters instead of the fourth order form described by Equation 2. The parameters of the optimized aspheric front surface of the inset lens are also depicted in Appendix A. The differences to the original design as given in Table 1 seem to be marginal but their impact on the performance is nevertheless significant. Another point to be considered in sensitivity analysis is the deviation in position of the image due to prismatic effects caused by tilting the surfaces. It must be ensured that the image will not be formed outside the fovea centralis with a diameter of approximately 1.5 mm. The image position was examined by tracing the chief ray through the optical system. Figure 15 shows the position of the chief ray in the image plane in dependence of the individual position tolerances of the freeform optics. Calculations of the chief ray positions were performed for three different adjustments of the refraction power: Far vision (−0.8 dpt) depicted by red symbols, intermediate distance (1.5 dpt) shown in blue, and near vision (3 dpt) labeled by green symbols.

Figure 15 shows that the prismatic effect of a tilt of the AH optics has no relevant impact on the image position on the fovea centralis. The major effect on the image position is caused by the wedge error with an adjustment of the refraction power of 3.0 dpt. In this case, the chief ray suffers a deviation in position of only 4 µm in *x*- and 7.1 µm in *y*-direction which ensures an image forming well inside the fovea centralis. The weak influence of the position errors on the image position is considered to be due to the small thickness of the AH-parts of only 500 µm.

## 5. Discussion

It is crucial in system integration to ensure the specified performance of the overall subsystem composed of individual components. This is due to the fact that each individual component has its own deviation from the nominal values and that the assembly process itself is subject to tolerances. Furthermore the operation of the system can affect the systems’ performance. Applying monolithic design approaches is a first step towards reducing assembly tolerances. To make sure that the manufactured subsystem fulfills its specification under operational conditions, a tolerance analysis can be carried out by means of a simulation tool. If the tolerance-afflicted system fails the system’s specification, a robust design approach with respect to an optimization of the functional surfaces may lead to a design fulfilling the specification even in the presence of fabrication and operational tolerances. The robust design approach presented in this article consists of three stages. The first step is a tolerance analysis: The relevant tolerances and their effects on the positioning of the optical components have to be determined. A subsequent sensitivity analysis gives information about the impact of the individual tolerances on the system’s performance. The next step consists of an optimization with respect to a robust design. The choice of the optimization parameters is of crucial importance to the success of the approach. The optimization parameters should describe the shape of the optics to be modified. The optimization criteria must represent the system’s specification, and restrictions can be used to ensure the manufacturability of the optimized design. The approach is finalized by a validation of the results by a comparative tolerance analysis.

This concept was applied to the optical micromachine of a demonstration model of an ophthalmic system. Design optimization was conducted using the shapes of the varifocal freeform surfaces as well as the shape of the aspherical inset lens of the rear housing as optimization parameters. This approach ensures the modular concept of the housing where the front and rear half-shells can be used for different active optical principles. Since modification of the freeform surfaces of both AH lens parts was allowed independently in optimization, the robust design results in surfaces which are no longer conjugated. As a consequence for manufacturing, this means that two individual molding tools have to be fabricated instead of only one which can be used for both lens parts in the case of the originally designed optics. This significantly increases tooling and potential process costs.

The nominal configuration was used as a reference for the evaluation of the robust design. However, it is in the nature of a tolerance optimization that the reference values cannot be reached for all tolerances under consideration. Therefore, a second criterion for evaluating the performance of the robust design plays an important role namely for avoiding the collapse of the performance. The robust design approach leads to important improvements in cases where a collapse of the imaging quality was observed already at very low spatial frequencies *i.e.*, for a mutual position error of the freeform lens parts perpendicular to the shifting direction (lateral position error) and a mutual inclination of the lens parts, the wedge error. In both cases, the robust design results in MTF curves which are smooth over the entire range under consideration. This improvement comes with a loss in imaging quality in the case of a total decentration of the optics perpendicular to the shifting direction of the lens parts (decentration of the total optics in y). Nevertheless, the MTF curve is also smooth in its course and suffers no abrupt drop in the frequency range considered. Lateral position error and decentration of the total optics in y are not independent of each other: optimization of the one error with respect to a robust design leads to a design more sensitive to the other error. Both errors have to be balanced to find a design offering the best minimum performance in a worst-case configuration. Since a poor imaging quality in the lower frequency range is not acceptable in the case of the AAS, the elimination of the collapse in MTF caused by the lateral position error and the wedge error as well as a smooth course of the MTF curve in the frequency range is essential. The robust design approach presented ensures a certain performance in the presence of assembly and manufacturing tolerances and helps to avoid disproportional efforts in assembly and manufacturing. To investigate the influence of the individual tolerances on the image position, tracing of the chief ray through the optical system was performed. The examination of the chief ray positions as a function of the individual tolerances showed only negligible effects and proved that image forming is well inside the fovea centralis.

## 6. Conclusions

System integration of complex microoptical systems is a challenging task the result of which is severely affected by yields on individual process steps or subsystems. In order to achieve a high yield on the final system, the yields from subsystem level down to individual process steps have to be maximized. At the same time, costs have to be kept in check, meaning that expensive measurement and active alignment procedures have to be reduced as much as possible. In this article a monolithic integration approach combined with design optimization for robustness were illustrated on the example of an ophthalmic implant. Fabrication and assembly tolerances of the optical micromachine were derived. The impact of the tolerances on the micromachine’s optical performance was analyzed by means of simulation. The simulation results indicated a severe impact on system’s performance due to the tolerances. In a subsequent step the freeform surfaces of the micromachine were optimized with respect to robustness to tolerances. The robust design ensures a satisfactory optical performance even in presence of fabrication and assembly tolerances. 

The article illustrates that the proposed two-pronged design approach leads to an optical subsystem that fulfills the specification while still achieving high yield with a reasonable effort.

## Figures and Tables

**Table 1:** Parameters of the freeform surfaces.

***a*_1_**	***a*_2_**	***a*_3_**	***a*_4_**	***a*_5_**	***a*_6_**	***a*_7_**
−3.18 × 10^−6^	−3.18 × 10^−5^	−1.48 × 10^−4^	0.00	5.14 × 10^−5^	−1.91 × 10^−5^	0.02
***a*_8_**	***a*_9_**	***a*_10_**	***a*_11_**	***a*_12_**	***a*_13_**	***a*_14_**
6.77 × 10^−5^	6.40 × 10^−3^	1.34 × 10^−4^	1.24 × 10^−4^	−8.92 × 10^−5^	−1.68 × 10^−5^	−1.24 × 10^−4^

**Table 2:** Parameters of the aspheric front surface of the inset lens.

***c***	***k***	***β*_1_**	***β*_2_**	***β*_3_**	***β*_4_**	***β*_5_**	***β*_6_**	***β*_7_**	***β*_8_**
0.738	−1.647	4.17 × 10^−4^	−0.328	−0.025	0.079	−0.039	0.010	−1.47 × 10^−3^	8.91 × 10^−5^

**Table 3:** Parameters of the aspheric surface of the optics integrated in the front half shell of the housing.

***c***	***k***	***β*_2_**	***β*_4_**	***β*_6_**
0.054	−2.444	0.015	1.08 × 10^−3^	−4.76 × 10^−5^

**Table 4:** Position errors due to a decentration of the total optics.

*x*-Direction	*y*-Direction	*z*-Direction
−46 µm–+52 µm	−45 µm–+48 µm	−50 µm–+50 µm

## Appendix A

The surface sag of the optimized freeform surfaces is given by Equation (A1). The parameters of the individual surfaces are given in Table A1 and Table A2. Numbering of the freeform surfaces is done from left to right following the beam path as shown in Figure 9.

*z*(*x,y*)*= a*_1_*x*^7^*+ a*_2_*x*^6^*y + a*_3_*x*^5^*y*^2^*+ a*_4_*x*^4^*y*^3^* + a*_5_*x*^3^*y*^4^* + a*_6_*x*^2^*y*^5^*+ a*_7_*xy*^6^*+ a*_8_*y*^7^*+ a*_9_*x*^6^*+ a*_10_*x*^5^*y + a*_11_*x*^4^*y*^2^*+a*_12_*x*^3^*y*^3^*+ a*_13_*x*^2^*y*^4^*+ a*_14_*xy*^5^*+ a*_15_*y*^6^*+ a*_16_*x*^5^*+ a*_17_*x*^4^*y*^1^*+ a*_18_*x*^3^*y*^2^*+ a*_19_*x*^2^*y*^3^*+ a*_20_*xy*^4^*+a*_21_*y*^5^*+ a*_22_*x*^4^*+ a*_23_*x*^3^*y + a*_24_*x*^2^*y*^2^*+ a*_25_*xy*^3^*+ a*_26_*y*^4^*+ a*_27_*x*^3^*+ a*_28_*x*^2^*y + a*_29_*xy^2^ + a*_30_*y*^3^*+ a*_31_*x*^2^*+a*_32_*xy + a*_33_*y*^2^*+ a*_34_*x + a*_35_*y* (A1)

**Table A1:** Parameters of freeform surface 1.

***a*_1_**	***a*_2_**	***a*_3_**	***a*_4_**	***a*_5_**	***a*_6_**	***a*_7_**	***a*_8_**	***a*_9_**
4.71 × 10^−6^	−1.46 × 10^−5^	−5.47 × 10^−5^	−7.50 × 10^−6^	1.50 × 10^−5^	1.99 × 10^−6^	−8.93 × 10^−6^	−2.63 × 10^−6^	3.16 × 10^−6^
***a*_10_**	***a*_11_**	***a*_12_**	***a*_13_**	***a*_14_**	***a*_15_**	***a*_16_**	***a*_17_**	***a*_18_**
2.78 × 10^−5^	8.46 × 10^−6^	−2.36 × 10^−5^	6.08 × 10^−5^	1.93 × 10^−5^	1.86 × 10^−5^	1.18 × 10^−4^	−7.58 × 10^−5^	2.03 × 10^−4^
***a*_19_**	***a*_20_**	***a*_21_**	***a*_22_**	***a*_23_**	***a*_24_**	***a*_25_**	***a*_26_**	***a*_27_**
−1.70 × 10^−4^	−1.05 × 10^−5^	−5.04 × 10^−6^	−8.57 × 10^−5^	−2.55 × 10^−4^	3.68 × 10^−5^	7.20 × 10^−5^	−2.26 × 10^−4^	−3.27 × 10^−3^
***a*_28_**	***a*_29_**	***a*_30_**	***a*_31_**	***a*_32_**	***a*_33_**	***a*_34_**	***a*_35_**	****
1.95 × 10^−2^	5.32 × 10^−4^	6.67 × 10^−3^	1.16 × 10^−3^	8.53 × 10^−4^	2.51 × 10^−3^	5.67 × 10^−5^	6.28 × 10^−4^	

**Table A2:** Parameters of freeform surface 2.

***a*_1_**	***a*_2_**	***a*_3_**	***a*_4_**	***a*_5_**	***a*_6_**	***a*_7_**	***a*_8_**	***a*_9_**
−3.06 × 10^−6^	−1.43 × 10^−5^	−6.84 × 10^−5^	1.21 × 10^−5^	−2.40 × 10^−6^	2.39 × 10^−5^	−8.91 × 10^−6^	1.01 × 10^−6^	−5.13 × 10^−5^
***a*_10_**	***a*_11_**	***a*_12_**	***a*_13_**	***a*_14_**	***a*_15_**	***a*_16_**	***a*_17_**	***a*_18_**
1.65 × 10^−5^	−1.24 × 10^−4^	−2.94 × 10^−5^	−8.11 × 10^−5^	5.29 × 10^−6^	−2.98 × 10^−5^	1.83 × 10^−4^	−1.21 × 10^−4^	2.92 × 10^−4^
***a*_19_**	***a*_20_**	***a*_21_**	***a*_22_**	***a*_23_**	***a*_24_**	***a*_25_**	***a*_26_**	***a*_27_**
−2.58 × 10^−4^	2.53 × 10^−5^	−3.19 × 10^−5^	2.93 × 10^−4^	−1.05 × 10^−4^	5.03 × 10^−4^	5.98 × 10^−5^	1.75 × 10^−4^	−3.46 × 10^−3^
***a*_28_**	***a*_29_**	***a*_30_**	***a*_31_**	***a*_32_**	***a*_33_**	***a*_34_**	***a*_35_**	****
1.98 × 10^−2^	4.23 × 10^−4^	6.74 × 10^−3^	−7.23 × 10^−4^	9.25 × 10^−4^	6.48 × 10^−4^	4.16 × 10^−4^	6.15 × 10^−4^	

The parameters of the optimized aspheric front surface of the inset lens are also depicted in Table A3.

**Table A3:** Parameters of the optimized aspheric front surface.

***c***	***k***	***β*_1_**	***β*_2_**	***β*_3_**	***β*_4_**	***β*_5_**	***β*_6_**	***β*_7_**	***β*_8_**
0.742	−1.690	4.47 × 10^−4^	−0.328	−0.025	0.079	−0.039	0.010	−1.47 × 10^−3^	7.49 × 10^−5^
